# Retraction: Cell Surface GRP78 Accelerated Breast Cancer Cell Proliferation and Migration by Activating STAT3

**DOI:** 10.1371/journal.pone.0284594

**Published:** 2023-04-11

**Authors:** The PLOS ONE Editors

Following the publication of this article [1], concerns were raised regarding the results presented in Figs 2, 4, and S2. Specifically,

The following panels appear similar:

- Lanes 1–3 of the Fig 2D p-PERK panel and lanes 2–4 of the Fig 2D IRE1α panel.
- The Fig 2D p-PERK panel of this study [1] and lanes 2–5 of the S3 Fig A AMPK panel of [2, retracted in 3].
- The IRE1α panel of this study [1] and lanes 1–4 of the S3 Fig A AMPK panel of [2, retracted in 3].
- Lanes 2–5 of the Fig 4A GRP78 panel of this study [1] and the Fig6B MCF7 p-STAT3 panel of [4, retracted in 5] when flipped horizontally.
- Lanes 3–6 of the Fig 4A p-STAT3 panel of this study [1] and the Fig 4A MCF7 p-AKT panel of [4, retracted in 5].
- The Fig 6D p-JAK2 panel of this study [1] and the Fig 5A PPARα panel of [6, corrected in 7] when flipped horizontally.
- The S2 Fig A p-STAT3 panel of this study [1] and lanes 1–3 of the Fig 7A MCF7 p-AKT panel of [4, retracted in 5].
- Lanes 2–3 of the S2 Fig A STAT3 panel of this study [1] and lanes 1–2 of the Fig 6A MCF7 STAT3 panel of [4, retracted in 5].

The authors did not respond to editorial requests for underlying data.

In light of the concerns affecting multiple figure panels that question the integrity of these data, the *PLOS ONE* Editors retract this article.

CW agreed with the retraction. XY, HL, XZ, LZ, XL, and SS either did not respond directly or could not be reached.

## References

[1] Yao X, Liu H, Zhang X, Zhang L, Li X, Wang C, et al. (2015) Cell Surface GRP78 Accelerated Breast Cancer Cell Proliferation and Migration by Activating STAT3. PLoS ONE 10(5): e0125634. 10.1371/journal.pone.0125634 25973748 PMC4431800

[2] Guan F, Ding Y, Zhang Y, Zhou Y, Li M, Wang C (2016) Curcumin Suppresses Proliferation and Migration of MDA-MB-231 Breast Cancer Cells through Autophagy-Dependent Akt Degradation. PLoS ONE 11(1): e0146553. 10.1371/journal.pone.0146553 26752181 PMC4708990

[3] The *PLOS ONE* Editors (2023) Retraction: Curcumin Suppresses Proliferation and Migration of MDA-MB-231 Breast Cancer Cells through Autophagy-Dependent Akt Degradation. PLoS ONE 18(3): e0283354. 10.1371/journal.pone.0283354 36920966 PMC10016713

[4] Ding Y, Cao Y, Wang B, Wang L, Zhang Y, Zhang D, et al. (2016) APPL1-Mediating Leptin Signaling Contributes to Proliferation and Migration of Cancer Cells. PLoS ONE 11(11): e0166172. 10.1371/journal.pone.0166172 27820851 PMC5098739

[5] The *PLOS ONE* Editors (2023) Retraction: APPL1-Mediating Leptin Signaling Contributes to Proliferation and Migration of Cancer Cells. PLoS ONE 18(3): e0283346. 10.1371/journal.pone.0283346 36920975 PMC10016670

[6] Zhang Ym., Li Mx., Tang Z. et al. Wogonin suppresses osteopontin expression in adipocytes by activating PPARα. Acta Pharmacol Sin 36, 987–997 (2015). 10.1038/aps.2015.37 26073326 PMC4564880

[7] Zhang Ym., Li Mx., Tang Z. et al. Author Correction: Wogonin suppresses osteopontin expression in adipocytes by activating PPARα. Acta Pharmacol Sin (2022). 10.1038/s41401-022-01034-x PMC10203130 36509903

